# Supplementary material for: Living on the edge: The sensitivity of arthropods to development and climate along an urban-wildland interface in the Sonoran Desert of central Arizona
Source: PLoS One. 2024 Apr 18;19(4):e0297227. doi: 10.1371/journal.pone.0297227 (PMC11025936; doi:10.1371/journal.pone.0297227)
Supplement: S1 File — Includes raw R-output along with csv files of raw data referenced in R-code. Arthropod data was obtained from [40]. (DOCX) [file pone.0297227.s004.docx]

McDowell Arthropods

Derek Uhey

#load libaries
library(betapart)
library(mvabund)
library(vegan)

## Loading required package: permute

## Loading required package: lattice

## This is vegan 2.5-6

library(janitor)

##
## Attaching package: 'janitor'

## The following objects are masked from 'package:stats':
##
## chisq.test, fisher.test

library(ggplot2)
library(dplyr)

##
## Attaching package: 'dplyr'

## The following objects are masked from 'package:stats':
##
## filter, lag

## The following objects are masked from 'package:base':
##
## intersect, setdiff, setequal, union

library(tidyr)
library(iNEXT)
library(DHARMa)

## This is DHARMa 0.4.4. For overview type '?DHARMa'. For recent changes, type news(package = 'DHARMa')

library(MuMIn)

## Warning: package 'MuMIn' was built under R version 4.0.5

##
## Attaching package: 'MuMIn'

## The following object is masked from 'package:mvabund':
##
## coefplot

library(emmeans)
library(glmmTMB)

## Warning: package 'glmmTMB' was built under R version 4.0.5

## Warning in checkMatrixPackageVersion(): Package version inconsistency detected.
## TMB was built with Matrix version 1.4.1
## Current Matrix version is 1.2.18
## Please re-install 'TMB' from source using install.packages('TMB', type = 'source') or ask CRAN for a binary version of 'TMB' matching CRAN's 'Matrix' package

## Warning in checkDepPackageVersion(dep_pkg = "TMB"): Package version inconsistency detected.
## glmmTMB was built with TMB version 1.8.0
## Current TMB version is 1.8.1
## Please re-install glmmTMB from source or restore original 'TMB' package (see '?reinstalling' for more information)

#Accumulation Curves

#LOAD DATASHEET: Average per date CSV
SonDate <- read.csv("Library/Mobile Documents/com~apple~CloudDocs/Academia/NAU/Research/PhD/SonoranCommunity/Sonoran_Wide_2020_AvDate_nomitesfliesspringtails.csv", header = TRUE)

#Select arthropods in columns 6:265
acc_comm = SonDate[,7:265]

#CREATE ACCUMULATION CURVES
curve_all = specaccum(acc_comm, method = "random", permutations = 999)
df.all <- data.frame(curve_all$richness, curve_all$sites, curve_all$sd)

#subset each habitat into its own df
SonDate %>% filter(Site == "Bell") -> Bell
SonDate %>% filter(Site == "Dixileta") -> Dixileta
SonDate %>% filter(Site == "Gateway") -> Gateway
SonDate %>% filter(Site == "LoneMtn") -> LoneMtn
SonDate %>% filter(Site == "Mine") -> Mine
SonDate %>% filter(Site == "Paraiso") -> Paraiso
SonDate %>% filter(Site == "Prospector") -> Prospector
SonDate %>% filter(Site == "Rincon") -> Rincon
SonDate %>% filter(Site == "Sunrise") -> Sunrise
SonDate %>% filter(Site == "TomThumb") -> TomThumb

#calc species accumulation curve for each habitat
curve_Bell = specaccum(Bell[, 7:265], method = "random", permutations = 999)
df.Bell <- data.frame(curve_Bell$richness, curve_Bell$sites, curve_Bell$sd)
curve_Dixileta = specaccum(Dixileta[, 7:265], method = "random", permutations = 999)
df.Dixileta <- data.frame(curve_Dixileta$richness, curve_Dixileta$sites, curve_Dixileta$sd)
curve_Gateway = specaccum(Gateway[, 7:265], method = "random", permutations = 999)
df.Gateway <- data.frame(curve_Gateway$richness, curve_Gateway$sites, curve_Gateway$sd)
curve_LoneMtn = specaccum(LoneMtn [, 7:265], method="random", permutations = 999)
df.LoneMtn <- data.frame(curve_LoneMtn $richness, curve_LoneMtn $sites, curve_LoneMtn $sd)
curve_Mine = specaccum(Mine[, 7:265], method = "random", permutations = 999)
df.Mine <- data.frame(curve_Mine$richness, curve_Mine$sites, curve_Mine$sd)
curve_Paraiso = specaccum(Paraiso[, 7:265], method = "random", permutations = 999)
df.Paraiso <- data.frame(curve_Paraiso$richness, curve_Paraiso$sites, curve_Paraiso$sd)
curve_Prospector = specaccum(Prospector[, 7:265], method = "random", permutations = 999)
df.Prospector <- data.frame(curve_Prospector$richness, curve_Prospector$sites, curve_Prospector$sd)
curve_Rincon = specaccum(Rincon[, 7:265], method = "random", permutations = 999)
df.Rincon <- data.frame(curve_Rincon$richness, curve_Rincon$sites, curve_Rincon$sd)
curve_Sunrise = specaccum(Sunrise[, 7:265], method = "random", permutations = 999)
df.Sunrise <- data.frame(curve_Sunrise$richness, curve_Sunrise$sites, curve_Sunrise$sd)
curve_TomThumb = specaccum(TomThumb[, 7:265], method = "random", permutations = 999)
df.TomThumb <- data.frame(curve_TomThumb$richness, curve_TomThumb$sites, curve_TomThumb$sd)

#make sure al new df have the same colnames
colnames(df.all) <- c("richness","sites","sd")
colnames(df.Bell) <- c("richness","sites","sd")
colnames(df.Dixileta) <- c("richness","sites","sd")
colnames(df.Gateway) <- c("richness","sites","sd")
colnames(df.LoneMtn) <- c("richness","sites","sd")
colnames(df.Mine) <- c("richness","sites","sd")
colnames(df.Paraiso) <- c("richness","sites","sd")
colnames(df.Prospector) <- c("richness","sites","sd")
colnames(df.Rincon) <- c("richness","sites","sd")
colnames(df.Sunrise) <- c("richness","sites","sd")
colnames(df.TomThumb) <- c("richness","sites","sd")

#adding new column to identify site type
df.all$type <- "All"
df.Bell$type <- "Bell"
df.Dixileta$type <- "Dixileta"
df.Gateway$type <- "Gateway"
df.LoneMtn $type <- "LoneMtn "
df.Mine$type <- "Mine"
df.Paraiso$type <- "Paraiso"
df.Prospector$type <- "Prospector"
df.Rincon$type <- "Rincon"
df.Sunrise$type <- "Sunrise"
df.TomThumb$type <- "TomThumb"

#rbinding all dfs together
df <- rbind(df.Bell,df.Dixileta,df.Gateway,df.LoneMtn,df.Mine,df.Paraiso,df.Prospector,df.Rincon,df.Sunrise,df.TomThumb)

#ggplotting species accumulation curves
ggplot() +
 geom_point(df, mapping=aes(x=sites, y=richness, color = type)) +
 geom_line(df, mapping=aes(x=sites, y=richness, color = type))


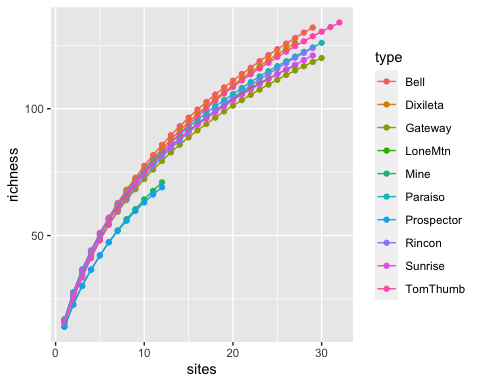


#BETAPART

#LOAD DATASHEET: Av per site, arthropods in columns 7:292
SonSite <- read.csv("Library/Mobile Documents/com~apple~CloudDocs/Academia/NAU/Research/PhD/SonoranCommunity/Sonoran_Wide_2020_AvSite.csv", header = TRUE)
SonS <-mvabund(SonSite[,2:287])
#betapart - Abundance
sites <- SonSite$Site
row.names(SonS) <- SonSite$Site
data.core <- betapart.core.abund(SonS)
data.multi <- beta.multi.abund(SonS)
data.samp <- beta.sample.abund(SonS, sites=10, samples=100)

##
 |
 | | 0%
 |
 |= | 1%
 |
 |= | 2%
 |
 |== | 3%
 |
 |=== | 4%
 |
 |==== | 5%
 |
 |==== | 6%
 |
 |===== | 7%
 |
 |====== | 8%
 |
 |====== | 9%
 |
 |======= | 10%
 |
 |======== | 11%
 |
 |======== | 12%
 |
 |========= | 13%
 |
 |========== | 14%
 |
 |========== | 15%
 |
 |=========== | 16%
 |
 |============ | 17%
 |
 |============= | 18%
 |
 |============= | 19%
 |
 |============== | 20%
 |
 |=============== | 21%
 |
 |=============== | 22%
 |
 |================ | 23%
 |
 |================= | 24%
 |
 |================== | 25%
 |
 |================== | 26%
 |
 |=================== | 27%
 |
 |==================== | 28%
 |
 |==================== | 29%
 |
 |===================== | 30%
 |
 |====================== | 31%
 |
 |====================== | 32%
 |
 |======================= | 33%
 |
 |======================== | 34%
 |
 |======================== | 35%
 |
 |========================= | 36%
 |
 |========================== | 37%
 |
 |=========================== | 38%
 |
 |=========================== | 39%
 |
 |============================ | 40%
 |
 |============================= | 41%
 |
 |============================= | 42%
 |
 |============================== | 43%
 |
 |=============================== | 44%
 |
 |================================ | 45%
 |
 |================================ | 46%
 |
 |================================= | 47%
 |
 |================================== | 48%
 |
 |================================== | 49%
 |
 |=================================== | 50%
 |
 |==================================== | 51%
 |
 |==================================== | 52%
 |
 |===================================== | 53%
 |
 |====================================== | 54%
 |
 |====================================== | 55%
 |
 |======================================= | 56%
 |
 |======================================== | 57%
 |
 |========================================= | 58%
 |
 |========================================= | 59%
 |
 |========================================== | 60%
 |
 |=========================================== | 61%
 |
 |=========================================== | 62%
 |
 |============================================ | 63%
 |
 |============================================= | 64%
 |
 |============================================== | 65%
 |
 |============================================== | 66%
 |
 |=============================================== | 67%
 |
 |================================================ | 68%
 |
 |================================================ | 69%
 |
 |================================================= | 70%
 |
 |================================================== | 71%
 |
 |================================================== | 72%
 |
 |=================================================== | 73%
 |
 |==================================================== | 74%
 |
 |==================================================== | 75%
 |
 |===================================================== | 76%
 |
 |====================================================== | 77%
 |
 |======================================================= | 78%
 |
 |======================================================= | 79%
 |
 |======================================================== | 80%
 |
 |========================================================= | 81%
 |
 |========================================================= | 82%
 |
 |========================================================== | 83%
 |
 |=========================================================== | 84%
 |
 |============================================================ | 85%
 |
 |============================================================ | 86%
 |
 |============================================================= | 87%
 |
 |============================================================== | 88%
 |
 |============================================================== | 89%
 |
 |=============================================================== | 90%
 |
 |================================================================ | 91%
 |
 |================================================================ | 92%
 |
 |================================================================= | 93%
 |
 |================================================================== | 94%
 |
 |================================================================== | 95%
 |
 |=================================================================== | 96%
 |
 |==================================================================== | 97%
 |
 |===================================================================== | 98%
 |
 |===================================================================== | 99%
 |
 |======================================================================| 100%

dist.data <- data.samp$sampled.values
beta.pair.abund(data.core)

## $beta.bray.bal
## Bell Dixileta Gateway LoneMtn Mine Paraiso
## Dixileta 0.2766770
## Gateway 0.3270645 0.3121955
## LoneMtn 0.3342201 0.3300999 0.3372269
## Mine 0.3916728 0.5123060 0.6300333 0.5065169
## Paraiso 0.4681595 0.4425807 0.3986050 0.3854252 0.5858649
## Prospector 0.4258975 0.3948369 0.3766137 0.4818135 0.4458047 0.4890923
## Rincon 0.4315172 0.4063787 0.2467320 0.4809562 0.6172003 0.4601581
## Sunrise 0.3670767 0.3204881 0.2994928 0.3622126 0.5049496 0.4601519
## TomThumb 0.3284829 0.2005407 0.2414089 0.4028125 0.5260887 0.3066805
## Prospector Rincon Sunrise
## Dixileta
## Gateway
## LoneMtn
## Mine
## Paraiso
## Prospector
## Rincon 0.4953604
## Sunrise 0.4364297 0.3045871
## TomThumb 0.3972815 0.4794803 0.3831277
##
## $beta.bray.gra
## Bell Dixileta Gateway LoneMtn Mine
## Dixileta 0.013848261
## Gateway 0.102021290 0.091372502
## LoneMtn 0.068850177 0.081939488 0.166410859
## Mine 0.235752153 0.181007700 0.092736880 0.232942199
## Paraiso 0.020973365 0.011318644 0.067864762 0.087434372 0.146400441
## Prospector 0.182971296 0.203216662 0.279674418 0.115365552 0.348372105
## Rincon 0.101614594 0.117072461 0.242279065 0.039838141 0.202731559
## Sunrise 0.043196796 0.059308438 0.152433467 0.022586032 0.219825433
## TomThumb 0.038537778 0.061119026 0.157174595 0.027648865 0.206270165
## Paraiso Prospector Rincon Sunrise
## Dixileta
## Gateway
## LoneMtn
## Mine
## Paraiso
## Prospector 0.180707262
## Rincon 0.116959588 0.074896820
## Sunrise 0.057977554 0.144289685 0.077790281
## TomThumb 0.066978772 0.160436420 0.063823885 0.006725907
##
## $beta.bray
## Bell Dixileta Gateway LoneMtn Mine Paraiso
## Dixileta 0.2905252
## Gateway 0.4290858 0.4035680
## LoneMtn 0.4030703 0.4120394 0.5036377
## Mine 0.6274250 0.6933137 0.7227702 0.7394591
## Paraiso 0.4891329 0.4538994 0.4664698 0.4728596 0.7322654
## Prospector 0.6088688 0.5980535 0.6562881 0.5971791 0.7941768 0.6697996
## Rincon 0.5331318 0.5234512 0.4890110 0.5207944 0.8199318 0.5771177
## Sunrise 0.4102735 0.3797966 0.4519263 0.3847986 0.7247750 0.5181294
## TomThumb 0.3670207 0.2616598 0.3985835 0.4304614 0.7323589 0.3736593
## Prospector Rincon Sunrise
## Dixileta
## Gateway
## LoneMtn
## Mine
## Paraiso
## Prospector
## Rincon 0.5702572
## Sunrise 0.5807194 0.3823774
## TomThumb 0.5577180 0.5433042 0.3898537

#ORDINATION
#LOAD DATASHEET: Av per date
SonDate <- read.csv("Library/Mobile Documents/com~apple~CloudDocs/Academia/NAU/Research/PhD/SonoranCommunity/Sonoran_Wide_2020_AvDate_nomitesfliesspringtails.csv", header = TRUE)
#Select arthropods in columns 6:265
SonD <-mvabund(SonDate[,11:265])

#Ordination for arthropods
ord<-metaMDS(SonD, distance = "bray",k=2, try=10, trymax=1000, autotransform=TRUE, expand=TRUE, plot=FALSE)

## Square root transformation
## Wisconsin double standardization
## Run 0 stress 0.3096562
## Run 1 stress 0.3091322
## ... New best solution
## ... Procrustes: rmse 0.04990186 max resid 0.1460036
## Run 2 stress 0.3107079
## Run 3 stress 0.3073704
## ... New best solution
## ... Procrustes: rmse 0.05317336 max resid 0.2009056
## Run 4 stress 0.311809
## Run 5 stress 0.3121676
## Run 6 stress 0.3143581
## Run 7 stress 0.310486
## Run 8 stress 0.3160642
## Run 9 stress 0.3061439
## ... New best solution
## ... Procrustes: rmse 0.04090626 max resid 0.1559765
## Run 10 stress 0.3102758
## Run 11 stress 0.3065573
## ... Procrustes: rmse 0.01744721 max resid 0.1473963
## Run 12 stress 0.3133053
## Run 13 stress 0.3076766
## Run 14 stress 0.311891
## Run 15 stress 0.3075837
## Run 16 stress 0.3074897
## Run 17 stress 0.3074696
## Run 18 stress 0.3084937
## Run 19 stress 0.3068396
## Run 20 stress 0.3073457
## Run 21 stress 0.3118717
## Run 22 stress 0.3074069
## Run 23 stress 0.306627
## ... Procrustes: rmse 0.01988364 max resid 0.1211332
## Run 24 stress 0.3075015
## Run 25 stress 0.3061713
## ... Procrustes: rmse 0.01853012 max resid 0.1508217
## Run 26 stress 0.3119115
## Run 27 stress 0.3067836
## Run 28 stress 0.3070101
## Run 29 stress 0.3147428
## Run 30 stress 0.3074432
## Run 31 stress 0.3066875
## Run 32 stress 0.3067104
## Run 33 stress 0.312936
## Run 34 stress 0.3070589
## Run 35 stress 0.3065779
## ... Procrustes: rmse 0.02359704 max resid 0.1667747
## Run 36 stress 0.3060444
## ... New best solution
## ... Procrustes: rmse 0.01753735 max resid 0.1559288
## Run 37 stress 0.3069739
## Run 38 stress 0.3090521
## Run 39 stress 0.3101372
## Run 40 stress 0.3075765
## Run 41 stress 0.3124516
## Run 42 stress 0.3067667
## Run 43 stress 0.3073473
## Run 44 stress 0.3065448
## Run 45 stress 0.3073227
## Run 46 stress 0.3101128
## Run 47 stress 0.3071732
## Run 48 stress 0.3090013
## Run 49 stress 0.3072329
## Run 50 stress 0.3064957
## ... Procrustes: rmse 0.03627099 max resid 0.1595622
## Run 51 stress 0.3072317
## Run 52 stress 0.3077255
## Run 53 stress 0.305813
## ... New best solution
## ... Procrustes: rmse 0.01497499 max resid 0.1539899
## Run 54 stress 0.3066981
## Run 55 stress 0.3076897
## Run 56 stress 0.3076604
## Run 57 stress 0.311042
## Run 58 stress 0.3080766
## Run 59 stress 0.307473
## Run 60 stress 0.3149446
## Run 61 stress 0.3066437
## Run 62 stress 0.3067303
## Run 63 stress 0.3092584
## Run 64 stress 0.3118028
## Run 65 stress 0.3063133
## Run 66 stress 0.3063506
## Run 67 stress 0.3098185
## Run 68 stress 0.3111211
## Run 69 stress 0.3064671
## Run 70 stress 0.3078221
## Run 71 stress 0.3091758
## Run 72 stress 0.3130896
## Run 73 stress 0.3068383
## Run 74 stress 0.3114485
## Run 75 stress 0.307739
## Run 76 stress 0.3066859
## Run 77 stress 0.3067779
## Run 78 stress 0.3066034
## Run 79 stress 0.3063663
## Run 80 stress 0.3066582
## Run 81 stress 0.3064099
## Run 82 stress 0.3065875
## Run 83 stress 0.3144716
## Run 84 stress 0.3066482
## Run 85 stress 0.3081184
## Run 86 stress 0.3089503
## Run 87 stress 0.3063714
## Run 88 stress 0.3101169
## Run 89 stress 0.3099142
## Run 90 stress 0.306748
## Run 91 stress 0.3069331
## Run 92 stress 0.3081818
## Run 93 stress 0.307023
## Run 94 stress 0.3070735
## Run 95 stress 0.3077694
## Run 96 stress 0.3076071
## Run 97 stress 0.3090835
## Run 98 stress 0.3178525
## Run 99 stress 0.3066277
## Run 100 stress 0.3108954
## Run 101 stress 0.3062717
## ... Procrustes: rmse 0.01730794 max resid 0.1552706
## Run 102 stress 0.3069558
## Run 103 stress 0.3088156
## Run 104 stress 0.3079579
## Run 105 stress 0.3073457
## Run 106 stress 0.3064701
## Run 107 stress 0.3067521
## Run 108 stress 0.3077133
## Run 109 stress 0.3128797
## Run 110 stress 0.3065206
## Run 111 stress 0.309251
## Run 112 stress 0.3077265
## Run 113 stress 0.3070074
## Run 114 stress 0.3079381
## Run 115 stress 0.3069498
## Run 116 stress 0.3066432
## Run 117 stress 0.3098096
## Run 118 stress 0.3071631
## Run 119 stress 0.3086097
## Run 120 stress 0.3132453
## Run 121 stress 0.3070621
## Run 122 stress 0.3066774
## Run 123 stress 0.306896
## Run 124 stress 0.3074183
## Run 125 stress 0.3068875
## Run 126 stress 0.3068774
## Run 127 stress 0.3064027
## Run 128 stress 0.3093487
## Run 129 stress 0.3078235
## Run 130 stress 0.3068741
## Run 131 stress 0.3097951
## Run 132 stress 0.3122319
## Run 133 stress 0.3107447
## Run 134 stress 0.30761
## Run 135 stress 0.3074781
## Run 136 stress 0.3074596
## Run 137 stress 0.3105321
## Run 138 stress 0.3090482
## Run 139 stress 0.3061751
## ... Procrustes: rmse 0.03162875 max resid 0.1556607
## Run 140 stress 0.308409
## Run 141 stress 0.3067655
## Run 142 stress 0.3098274
## Run 143 stress 0.307412
## Run 144 stress 0.3064221
## Run 145 stress 0.3121108
## Run 146 stress 0.3069501
## Run 147 stress 0.312133
## Run 148 stress 0.3071629
## Run 149 stress 0.3067122
## Run 150 stress 0.3106123
## Run 151 stress 0.3068502
## Run 152 stress 0.3133653
## Run 153 stress 0.3072276
## Run 154 stress 0.3077135
## Run 155 stress 0.3083151
## Run 156 stress 0.3069673
## Run 157 stress 0.307574
## Run 158 stress 0.306716
## Run 159 stress 0.3119511
## Run 160 stress 0.306752
## Run 161 stress 0.3071504
## Run 162 stress 0.3076875
## Run 163 stress 0.3067997
## Run 164 stress 0.3069718
## Run 165 stress 0.3063664
## Run 166 stress 0.3067379
## Run 167 stress 0.3117492
## Run 168 stress 0.3106235
## Run 169 stress 0.3061097
## ... Procrustes: rmse 0.01813804 max resid 0.1568152
## Run 170 stress 0.3117836
## Run 171 stress 0.3107017
## Run 172 stress 0.3067998
## Run 173 stress 0.3059011
## ... Procrustes: rmse 0.01286794 max resid 0.1170795
## Run 174 stress 0.3084367
## Run 175 stress 0.3075553
## Run 176 stress 0.3095279
## Run 177 stress 0.3099214
## Run 178 stress 0.3059563
## ... Procrustes: rmse 0.01799227 max resid 0.1175584
## Run 179 stress 0.3067085
## Run 180 stress 0.3067632
## Run 181 stress 0.3068607
## Run 182 stress 0.308105
## Run 183 stress 0.3105351
## Run 184 stress 0.3077602
## Run 185 stress 0.3066363
## Run 186 stress 0.3077071
## Run 187 stress 0.3072837
## Run 188 stress 0.306327
## Run 189 stress 0.306576
## Run 190 stress 0.3068883
## Run 191 stress 0.30745
## Run 192 stress 0.3066723
## Run 193 stress 0.3073817
## Run 194 stress 0.3083311
## Run 195 stress 0.3075123
## Run 196 stress 0.3082615
## Run 197 stress 0.3106472
## Run 198 stress 0.3066429
## Run 199 stress 0.3070036
## Run 200 stress 0.3088756
## Run 201 stress 0.3070134
## Run 202 stress 0.312173
## Run 203 stress 0.3098623
## Run 204 stress 0.3123515
## Run 205 stress 0.3072321
## Run 206 stress 0.3118114
## Run 207 stress 0.3078707
## Run 208 stress 0.3083684
## Run 209 stress 0.3099096
## Run 210 stress 0.3067625
## Run 211 stress 0.3062643
## ... Procrustes: rmse 0.01711617 max resid 0.1156687
## Run 212 stress 0.307317
## Run 213 stress 0.3082481
## Run 214 stress 0.3102022
## Run 215 stress 0.3064336
## Run 216 stress 0.3156815
## Run 217 stress 0.3105704
## Run 218 stress 0.3081877
## Run 219 stress 0.3067333
## Run 220 stress 0.3069874
## Run 221 stress 0.3105835
## Run 222 stress 0.3067221
## Run 223 stress 0.3066724
## Run 224 stress 0.3066256
## Run 225 stress 0.3063674
## Run 226 stress 0.3067099
## Run 227 stress 0.3085876
## Run 228 stress 0.3071315
## Run 229 stress 0.3124138
## Run 230 stress 0.3062865
## ... Procrustes: rmse 0.01649775 max resid 0.1796581
## Run 231 stress 0.3122976
## Run 232 stress 0.3083932
## Run 233 stress 0.3078694
## Run 234 stress 0.3075952
## Run 235 stress 0.3139999
## Run 236 stress 0.3083972
## Run 237 stress 0.3064633
## Run 238 stress 0.3141955
## Run 239 stress 0.3062523
## ... Procrustes: rmse 0.03499961 max resid 0.1669757
## Run 240 stress 0.3064708
## Run 241 stress 0.3067697
## Run 242 stress 0.3107716
## Run 243 stress 0.3101812
## Run 244 stress 0.3115064
## Run 245 stress 0.3070691
## Run 246 stress 0.3083637
## Run 247 stress 0.30696
## Run 248 stress 0.3069009
## Run 249 stress 0.3075717
## Run 250 stress 0.3071234
## Run 251 stress 0.3081052
## Run 252 stress 0.3068365
## Run 253 stress 0.307204
## Run 254 stress 0.3093593
## Run 255 stress 0.307472
## Run 256 stress 0.3099614
## Run 257 stress 0.3088189
## Run 258 stress 0.3073862
## Run 259 stress 0.3104122
## Run 260 stress 0.3137112
## Run 261 stress 0.311673
## Run 262 stress 0.3092841
## Run 263 stress 0.3113594
## Run 264 stress 0.3071941
## Run 265 stress 0.311683
## Run 266 stress 0.3131122
## Run 267 stress 0.3118604
## Run 268 stress 0.3121661
## Run 269 stress 0.3068112
## Run 270 stress 0.3073203
## Run 271 stress 0.3077472
## Run 272 stress 0.3075777
## Run 273 stress 0.3098175
## Run 274 stress 0.3090516
## Run 275 stress 0.3075248
## Run 276 stress 0.3096586
## Run 277 stress 0.3073985
## Run 278 stress 0.3086429
## Run 279 stress 0.3067932
## Run 280 stress 0.3136747
## Run 281 stress 0.3084592
## Run 282 stress 0.3085134
## Run 283 stress 0.3111137
## Run 284 stress 0.3071015
## Run 285 stress 0.3164533
## Run 286 stress 0.3066814
## Run 287 stress 0.3075157
## Run 288 stress 0.3064487
## Run 289 stress 0.3077576
## Run 290 stress 0.3081425
## Run 291 stress 0.3105513
## Run 292 stress 0.3061919
## ... Procrustes: rmse 0.01699945 max resid 0.1170628
## Run 293 stress 0.3103852
## Run 294 stress 0.307262
## Run 295 stress 0.3069837
## Run 296 stress 0.3065394
## Run 297 stress 0.3061951
## ... Procrustes: rmse 0.01391596 max resid 0.1166712
## Run 298 stress 0.310114
## Run 299 stress 0.3091601
## Run 300 stress 0.3095553
## Run 301 stress 0.3062318
## ... Procrustes: rmse 0.0255265 max resid 0.1722384
## Run 302 stress 0.3067782
## Run 303 stress 0.3085739
## Run 304 stress 0.3107117
## Run 305 stress 0.3075845
## Run 306 stress 0.3069949
## Run 307 stress 0.3121501
## Run 308 stress 0.3144402
## Run 309 stress 0.3078143
## Run 310 stress 0.3073603
## Run 311 stress 0.309173
## Run 312 stress 0.307642
## Run 313 stress 0.3111291
## Run 314 stress 0.307597
## Run 315 stress 0.3062018
## ... Procrustes: rmse 0.01985246 max resid 0.1561626
## Run 316 stress 0.3076013
## Run 317 stress 0.309396
## Run 318 stress 0.3078349
## Run 319 stress 0.3066144
## Run 320 stress 0.3059743
## ... Procrustes: rmse 0.01396997 max resid 0.1164671
## Run 321 stress 0.3101674
## Run 322 stress 0.3070173
## Run 323 stress 0.3073182
## Run 324 stress 0.3085545
## Run 325 stress 0.3063158
## Run 326 stress 0.307575
## Run 327 stress 0.306632
## Run 328 stress 0.315242
## Run 329 stress 0.3069578
## Run 330 stress 0.3063745
## Run 331 stress 0.3072299
## Run 332 stress 0.3138324
## Run 333 stress 0.3064724
## Run 334 stress 0.3074953
## Run 335 stress 0.30724
## Run 336 stress 0.3077737
## Run 337 stress 0.3113035
## Run 338 stress 0.3062579
## ... Procrustes: rmse 0.03271237 max resid 0.1681913
## Run 339 stress 0.3115719
## Run 340 stress 0.3065558
## Run 341 stress 0.3144931
## Run 342 stress 0.3064161
## Run 343 stress 0.3097844
## Run 344 stress 0.3103729
## Run 345 stress 0.3068418
## Run 346 stress 0.306334
## Run 347 stress 0.3089372
## Run 348 stress 0.3072034
## Run 349 stress 0.30734
## Run 350 stress 0.3065706
## Run 351 stress 0.3066061
## Run 352 stress 0.3073077
## Run 353 stress 0.306078
## ... Procrustes: rmse 0.03416269 max resid 0.1596963
## Run 354 stress 0.3070612
## Run 355 stress 0.3079639
## Run 356 stress 0.3061811
## ... Procrustes: rmse 0.01536016 max resid 0.1159191
## Run 357 stress 0.3070765
## Run 358 stress 0.3118226
## Run 359 stress 0.309529
## Run 360 stress 0.3106927
## Run 361 stress 0.3069313
## Run 362 stress 0.3076272
## Run 363 stress 0.3071377
## Run 364 stress 0.3089196
## Run 365 stress 0.3075046
## Run 366 stress 0.3074263
## Run 367 stress 0.306212
## ... Procrustes: rmse 0.01589248 max resid 0.1168562
## Run 368 stress 0.3093378
## Run 369 stress 0.3067494
## Run 370 stress 0.3077022
## Run 371 stress 0.3070271
## Run 372 stress 0.3063187
## Run 373 stress 0.3086927
## Run 374 stress 0.3071738
## Run 375 stress 0.3112593
## Run 376 stress 0.3113926
## Run 377 stress 0.3103171
## Run 378 stress 0.3095156
## Run 379 stress 0.3068857
## Run 380 stress 0.306861
## Run 381 stress 0.3064333
## Run 382 stress 0.3071807
## Run 383 stress 0.306388
## Run 384 stress 0.3081143
## Run 385 stress 0.3065138
## Run 386 stress 0.3073615
## Run 387 stress 0.3080555
## Run 388 stress 0.3158005
## Run 389 stress 0.3076339
## Run 390 stress 0.3076416
## Run 391 stress 0.3067788
## Run 392 stress 0.3067174
## Run 393 stress 0.3091141
## Run 394 stress 0.3068683
## Run 395 stress 0.3068653
## Run 396 stress 0.3088001
## Run 397 stress 0.3090328
## Run 398 stress 0.3111924
## Run 399 stress 0.3150538
## Run 400 stress 0.3069271
## Run 401 stress 0.309996
## Run 402 stress 0.3128411
## Run 403 stress 0.3088719
## Run 404 stress 0.3099377
## Run 405 stress 0.307487
## Run 406 stress 0.3124722
## Run 407 stress 0.3074055
## Run 408 stress 0.3145021
## Run 409 stress 0.3097773
## Run 410 stress 0.3135798
## Run 411 stress 0.3067073
## Run 412 stress 0.3091205
## Run 413 stress 0.3066876
## Run 414 stress 0.3115517
## Run 415 stress 0.3066418
## Run 416 stress 0.3064669
## Run 417 stress 0.3064729
## Run 418 stress 0.3071073
## Run 419 stress 0.3099332
## Run 420 stress 0.3070314
## Run 421 stress 0.3065062
## Run 422 stress 0.3106243
## Run 423 stress 0.3065134
## Run 424 stress 0.3069847
## Run 425 stress 0.3118454
## Run 426 stress 0.308074
## Run 427 stress 0.3082191
## Run 428 stress 0.3077268
## Run 429 stress 0.3103064
## Run 430 stress 0.3067296
## Run 431 stress 0.3076517
## Run 432 stress 0.3118021
## Run 433 stress 0.307494
## Run 434 stress 0.3065037
## Run 435 stress 0.3074325
## Run 436 stress 0.3086195
## Run 437 stress 0.3064253
## Run 438 stress 0.306943
## Run 439 stress 0.3087338
## Run 440 stress 0.3099804
## Run 441 stress 0.3067666
## Run 442 stress 0.3099749
## Run 443 stress 0.3073001
## Run 444 stress 0.3078302
## Run 445 stress 0.3127482
## Run 446 stress 0.3086369
## Run 447 stress 0.310758
## Run 448 stress 0.3068696
## Run 449 stress 0.3102353
## Run 450 stress 0.3078734
## Run 451 stress 0.3085227
## Run 452 stress 0.3074321
## Run 453 stress 0.3066911
## Run 454 stress 0.3062307
## ... Procrustes: rmse 0.01994964 max resid 0.1181111
## Run 455 stress 0.3095974
## Run 456 stress 0.3101068
## Run 457 stress 0.3070247
## Run 458 stress 0.3071354
## Run 459 stress 0.3086985
## Run 460 stress 0.3065487
## Run 461 stress 0.3118664
## Run 462 stress 0.3085503
## Run 463 stress 0.3082703
## Run 464 stress 0.3076461
## Run 465 stress 0.3101124
## Run 466 stress 0.3075445
## Run 467 stress 0.3106276
## Run 468 stress 0.3110991
## Run 469 stress 0.3068697
## Run 470 stress 0.3067594
## Run 471 stress 0.3077519
## Run 472 stress 0.3075386
## Run 473 stress 0.3102295
## Run 474 stress 0.3091602
## Run 475 stress 0.3087797
## Run 476 stress 0.3066581
## Run 477 stress 0.3082607
## Run 478 stress 0.3096087
## Run 479 stress 0.3064686
## Run 480 stress 0.3067254
## Run 481 stress 0.3069922
## Run 482 stress 0.3067759
## Run 483 stress 0.307326
## Run 484 stress 0.3065778
## Run 485 stress 0.3089003
## Run 486 stress 0.3071856
## Run 487 stress 0.3082996
## Run 488 stress 0.3078519
## Run 489 stress 0.3109979
## Run 490 stress 0.3062277
## ... Procrustes: rmse 0.02029978 max resid 0.1539018
## Run 491 stress 0.3068131
## Run 492 stress 0.3101242
## Run 493 stress 0.3067649
## Run 494 stress 0.3090994
## Run 495 stress 0.3080016
## Run 496 stress 0.307748
## Run 497 stress 0.3065888
## Run 498 stress 0.3100075
## Run 499 stress 0.3081158
## Run 500 stress 0.3070284
## Run 501 stress 0.3088666
## Run 502 stress 0.306208
## ... Procrustes: rmse 0.01799465 max resid 0.1683533
## Run 503 stress 0.3111503
## Run 504 stress 0.3073932
## Run 505 stress 0.3072361
## Run 506 stress 0.308046
## Run 507 stress 0.3070817
## Run 508 stress 0.3068108
## Run 509 stress 0.3083295
## Run 510 stress 0.310789
## Run 511 stress 0.3105203
## Run 512 stress 0.3100885
## Run 513 stress 0.3081826
## Run 514 stress 0.3132399
## Run 515 stress 0.3066038
## Run 516 stress 0.3091417
## Run 517 stress 0.3097395
## Run 518 stress 0.3065487
## Run 519 stress 0.3080706
## Run 520 stress 0.3065617
## Run 521 stress 0.3092774
## Run 522 stress 0.3104013
## Run 523 stress 0.3067031
## Run 524 stress 0.3076537
## Run 525 stress 0.3099966
## Run 526 stress 0.3064424
## Run 527 stress 0.3116238
## Run 528 stress 0.3101621
## Run 529 stress 0.3119413
## Run 530 stress 0.3064234
## Run 531 stress 0.308727
## Run 532 stress 0.306372
## Run 533 stress 0.3079136
## Run 534 stress 0.3064979
## Run 535 stress 0.3076537
## Run 536 stress 0.3101983
## Run 537 stress 0.3070345
## Run 538 stress 0.3066764
## Run 539 stress 0.311782
## Run 540 stress 0.3059171
## ... Procrustes: rmse 0.01310663 max resid 0.1134421
## Run 541 stress 0.3112195
## Run 542 stress 0.307507
## Run 543 stress 0.3100823
## Run 544 stress 0.3065597
## Run 545 stress 0.3107148
## Run 546 stress 0.3068518
## Run 547 stress 0.3072653
## Run 548 stress 0.3123052
## Run 549 stress 0.3077802
## Run 550 stress 0.3101446
## Run 551 stress 0.3091408
## Run 552 stress 0.3080467
## Run 553 stress 0.3068795
## Run 554 stress 0.3152003
## Run 555 stress 0.3072994
## Run 556 stress 0.314858
## Run 557 stress 0.3067209
## Run 558 stress 0.3074627
## Run 559 stress 0.3078516
## Run 560 stress 0.3064973
## Run 561 stress 0.3068062
## Run 562 stress 0.3065015
## Run 563 stress 0.3071862
## Run 564 stress 0.3083403
## Run 565 stress 0.3063103
## ... Procrustes: rmse 0.02113313 max resid 0.1750225
## Run 566 stress 0.3075211
## Run 567 stress 0.3080708
## Run 568 stress 0.307502
## Run 569 stress 0.3141777
## Run 570 stress 0.3059424
## ... Procrustes: rmse 0.01373981 max resid 0.1157602
## Run 571 stress 0.3092181
## Run 572 stress 0.3103018
## Run 573 stress 0.3097646
## Run 574 stress 0.3076323
## Run 575 stress 0.3066637
## Run 576 stress 0.3123335
## Run 577 stress 0.3091092
## Run 578 stress 0.3081636
## Run 579 stress 0.3070462
## Run 580 stress 0.3074043
## Run 581 stress 0.3116725
## Run 582 stress 0.3080379
## Run 583 stress 0.3113575
## Run 584 stress 0.3069049
## Run 585 stress 0.3079913
## Run 586 stress 0.3108248
## Run 587 stress 0.3072466
## Run 588 stress 0.3106891
## Run 589 stress 0.3097328
## Run 590 stress 0.3100701
## Run 591 stress 0.309061
## Run 592 stress 0.3070349
## Run 593 stress 0.3066608
## Run 594 stress 0.308094
## Run 595 stress 0.3071936
## Run 596 stress 0.3064026
## Run 597 stress 0.3066809
## Run 598 stress 0.3062188
## ... Procrustes: rmse 0.02369935 max resid 0.149656
## Run 599 stress 0.3065452
## Run 600 stress 0.3067332
## Run 601 stress 0.3068885
## Run 602 stress 0.3071871
## Run 603 stress 0.3071386
## Run 604 stress 0.3070484
## Run 605 stress 0.3086822
## Run 606 stress 0.310434
## Run 607 stress 0.3079522
## Run 608 stress 0.3063485
## Run 609 stress 0.3073885
## Run 610 stress 0.3080407
## Run 611 stress 0.3143697
## Run 612 stress 0.3085217
## Run 613 stress 0.3136276
## Run 614 stress 0.3064185
## Run 615 stress 0.3078655
## Run 616 stress 0.3065132
## Run 617 stress 0.3121002
## Run 618 stress 0.3075055
## Run 619 stress 0.3104671
## Run 620 stress 0.3067896
## Run 621 stress 0.3131096
## Run 622 stress 0.3076724
## Run 623 stress 0.3087672
## Run 624 stress 0.3066101
## Run 625 stress 0.307355
## Run 626 stress 0.3068004
## Run 627 stress 0.3108036
## Run 628 stress 0.3075578
## Run 629 stress 0.3073914
## Run 630 stress 0.3063962
## Run 631 stress 0.3102948
## Run 632 stress 0.3068844
## Run 633 stress 0.3119883
## Run 634 stress 0.3070354
## Run 635 stress 0.3093778
## Run 636 stress 0.3088292
## Run 637 stress 0.3098578
## Run 638 stress 0.3081341
## Run 639 stress 0.3083447
## Run 640 stress 0.3068581
## Run 641 stress 0.3066326
## Run 642 stress 0.3085182
## Run 643 stress 0.3073162
## Run 644 stress 0.3063409
## Run 645 stress 0.3066382
## Run 646 stress 0.3074914
## Run 647 stress 0.3083978
## Run 648 stress 0.3097487
## Run 649 stress 0.3064833
## Run 650 stress 0.3079219
## Run 651 stress 0.3070292
## Run 652 stress 0.3073397
## Run 653 stress 0.3084053
## Run 654 stress 0.3082191
## Run 655 stress 0.3064431
## Run 656 stress 0.3118204
## Run 657 stress 0.3121912
## Run 658 stress 0.3069383
## Run 659 stress 0.3111761
## Run 660 stress 0.3072735
## Run 661 stress 0.3088013
## Run 662 stress 0.3088703
## Run 663 stress 0.3068045
## Run 664 stress 0.3070593
## Run 665 stress 0.307755
## Run 666 stress 0.3074752
## Run 667 stress 0.30902
## Run 668 stress 0.312094
## Run 669 stress 0.3069335
## Run 670 stress 0.3080112
## Run 671 stress 0.30684
## Run 672 stress 0.306544
## Run 673 stress 0.3094829
## Run 674 stress 0.3089885
## Run 675 stress 0.3066697
## Run 676 stress 0.3086229
## Run 677 stress 0.3070342
## Run 678 stress 0.3089198
## Run 679 stress 0.3079427
## Run 680 stress 0.3152302
## Run 681 stress 0.3067248
## Run 682 stress 0.3105545
## Run 683 stress 0.3070969
## Run 684 stress 0.3067976
## Run 685 stress 0.3084127
## Run 686 stress 0.3078167
## Run 687 stress 0.3073344
## Run 688 stress 0.3079788
## Run 689 stress 0.3066243
## Run 690 stress 0.3067458
## Run 691 stress 0.3074292
## Run 692 stress 0.3125346
## Run 693 stress 0.3106298
## Run 694 stress 0.3095985
## Run 695 stress 0.309781
## Run 696 stress 0.3062239
## ... Procrustes: rmse 0.02593631 max resid 0.1744124
## Run 697 stress 0.3069464
## Run 698 stress 0.3113165
## Run 699 stress 0.3093214
## Run 700 stress 0.3115904
## Run 701 stress 0.30588
## ... Procrustes: rmse 0.01607567 max resid 0.1172845
## Run 702 stress 0.3067645
## Run 703 stress 0.3086364
## Run 704 stress 0.3115522
## Run 705 stress 0.3063463
## Run 706 stress 0.3099095
## Run 707 stress 0.306368
## Run 708 stress 0.3073913
## Run 709 stress 0.3084627
## Run 710 stress 0.3093398
## Run 711 stress 0.3067975
## Run 712 stress 0.3104885
## Run 713 stress 0.3092172
## Run 714 stress 0.306821
## Run 715 stress 0.30657
## Run 716 stress 0.3070582
## Run 717 stress 0.310212
## Run 718 stress 0.3070867
## Run 719 stress 0.3073913
## Run 720 stress 0.3074712
## Run 721 stress 0.3099419
## Run 722 stress 0.3114895
## Run 723 stress 0.3080766
## Run 724 stress 0.3071064
## Run 725 stress 0.3068816
## Run 726 stress 0.3067873
## Run 727 stress 0.3105399
## Run 728 stress 0.3073598
## Run 729 stress 0.3081781
## Run 730 stress 0.3075484
## Run 731 stress 0.3101104
## Run 732 stress 0.307006
## Run 733 stress 0.3133488
## Run 734 stress 0.307468
## Run 735 stress 0.3069305
## Run 736 stress 0.3105746
## Run 737 stress 0.3097353
## Run 738 stress 0.3073168
## Run 739 stress 0.3068882
## Run 740 stress 0.3072403
## Run 741 stress 0.3067131
## Run 742 stress 0.310097
## Run 743 stress 0.3068577
## Run 744 stress 0.3106315
## Run 745 stress 0.3073726
## Run 746 stress 0.3076296
## Run 747 stress 0.3063291
## Run 748 stress 0.3067774
## Run 749 stress 0.3069728
## Run 750 stress 0.3073186
## Run 751 stress 0.311688
## Run 752 stress 0.3068702
## Run 753 stress 0.3071352
## Run 754 stress 0.3065622
## Run 755 stress 0.3125754
## Run 756 stress 0.3156548
## Run 757 stress 0.3106642
## Run 758 stress 0.3075953
## Run 759 stress 0.3105443
## Run 760 stress 0.306982
## Run 761 stress 0.3066792
## Run 762 stress 0.3083001
## Run 763 stress 0.3115244
## Run 764 stress 0.3098418
## Run 765 stress 0.3088911
## Run 766 stress 0.306956
## Run 767 stress 0.3074089
## Run 768 stress 0.307658
## Run 769 stress 0.3100013
## Run 770 stress 0.3067517
## Run 771 stress 0.3113851
## Run 772 stress 0.3071799
## Run 773 stress 0.3095612
## Run 774 stress 0.3110327
## Run 775 stress 0.3095439
## Run 776 stress 0.3067169
## Run 777 stress 0.306484
## Run 778 stress 0.3063128
## ... Procrustes: rmse 0.02361153 max resid 0.1581632
## Run 779 stress 0.3068127
## Run 780 stress 0.3069764
## Run 781 stress 0.315529
## Run 782 stress 0.3067627
## Run 783 stress 0.3064983
## Run 784 stress 0.3067229
## Run 785 stress 0.3078207
## Run 786 stress 0.306485
## Run 787 stress 0.3064528
## Run 788 stress 0.3138911
## Run 789 stress 0.306385
## Run 790 stress 0.3067392
## Run 791 stress 0.3067949
## Run 792 stress 0.307706
## Run 793 stress 0.3068262
## Run 794 stress 0.3070603
## Run 795 stress 0.3145688
## Run 796 stress 0.3068765
## Run 797 stress 0.3066766
## Run 798 stress 0.3143794
## Run 799 stress 0.3079334
## Run 800 stress 0.3109301
## Run 801 stress 0.3072615
## Run 802 stress 0.3072567
## Run 803 stress 0.307271
## Run 804 stress 0.3066141
## Run 805 stress 0.3085123
## Run 806 stress 0.3060979
## ... Procrustes: rmse 0.03226653 max resid 0.160442
## Run 807 stress 0.3083204
## Run 808 stress 0.3089472
## Run 809 stress 0.3070522
## Run 810 stress 0.3068688
## Run 811 stress 0.3077293
## Run 812 stress 0.3073774
## Run 813 stress 0.3125131
## Run 814 stress 0.3073572
## Run 815 stress 0.3072236
## Run 816 stress 0.3091888
## Run 817 stress 0.3066603
## Run 818 stress 0.3125507
## Run 819 stress 0.307107
## Run 820 stress 0.3068484
## Run 821 stress 0.3070518
## Run 822 stress 0.3099899
## Run 823 stress 0.3064218
## Run 824 stress 0.3075558
## Run 825 stress 0.3087967
## Run 826 stress 0.3096348
## Run 827 stress 0.3067016
## Run 828 stress 0.3117115
## Run 829 stress 0.3067038
## Run 830 stress 0.3079178
## Run 831 stress 0.3059501
## ... Procrustes: rmse 0.01448599 max resid 0.1141108
## Run 832 stress 0.3122293
## Run 833 stress 0.3071673
## Run 834 stress 0.3084035
## Run 835 stress 0.3068571
## Run 836 stress 0.3109194
## Run 837 stress 0.313831
## Run 838 stress 0.3061389
## ... Procrustes: rmse 0.02105765 max resid 0.155064
## Run 839 stress 0.3094777
## Run 840 stress 0.3071144
## Run 841 stress 0.3081641
## Run 842 stress 0.3088927
## Run 843 stress 0.3069971
## Run 844 stress 0.312595
## Run 845 stress 0.307949
## Run 846 stress 0.3127788
## Run 847 stress 0.3133864
## Run 848 stress 0.3093134
## Run 849 stress 0.3095706
## Run 850 stress 0.3100981
## Run 851 stress 0.3088998
## Run 852 stress 0.3075846
## Run 853 stress 0.3076158
## Run 854 stress 0.3128213
## Run 855 stress 0.3070004
## Run 856 stress 0.3061606
## ... Procrustes: rmse 0.01733439 max resid 0.1188589
## Run 857 stress 0.3068343
## Run 858 stress 0.3080079
## Run 859 stress 0.3083016
## Run 860 stress 0.3102021
## Run 861 stress 0.3066976
## Run 862 stress 0.3073923
## Run 863 stress 0.3100168
## Run 864 stress 0.3063559
## Run 865 stress 0.3138495
## Run 866 stress 0.3071727
## Run 867 stress 0.3069462
## Run 868 stress 0.3082588
## Run 869 stress 0.3080552
## Run 870 stress 0.3075819
## Run 871 stress 0.3089225
## Run 872 stress 0.3095757
## Run 873 stress 0.3075898
## Run 874 stress 0.3089475
## Run 875 stress 0.3090844
## Run 876 stress 0.3103964
## Run 877 stress 0.3100721
## Run 878 stress 0.3104919
## Run 879 stress 0.3066122
## Run 880 stress 0.3071554
## Run 881 stress 0.3107924
## Run 882 stress 0.3066305
## Run 883 stress 0.3084759
## Run 884 stress 0.3106546
## Run 885 stress 0.3081236
## Run 886 stress 0.3073258
## Run 887 stress 0.3063282
## Run 888 stress 0.3077267
## Run 889 stress 0.3066739
## Run 890 stress 0.31082
## Run 891 stress 0.3106744
## Run 892 stress 0.3072937
## Run 893 stress 0.3070121
## Run 894 stress 0.3129797
## Run 895 stress 0.3074395
## Run 896 stress 0.3132015
## Run 897 stress 0.3092985
## Run 898 stress 0.3064677
## Run 899 stress 0.3065515
## Run 900 stress 0.3084124
## Run 901 stress 0.3116196
## Run 902 stress 0.3089428
## Run 903 stress 0.3102164
## Run 904 stress 0.3059535
## ... Procrustes: rmse 0.01580876 max resid 0.1571551
## Run 905 stress 0.3084309
## Run 906 stress 0.3088043
## Run 907 stress 0.3066935
## Run 908 stress 0.3107689
## Run 909 stress 0.3070641
## Run 910 stress 0.3075413
## Run 911 stress 0.3095395
## Run 912 stress 0.3065288
## Run 913 stress 0.3073444
## Run 914 stress 0.3069706
## Run 915 stress 0.3068826
## Run 916 stress 0.3075205
## Run 917 stress 0.3162597
## Run 918 stress 0.3064603
## Run 919 stress 0.3112475
## Run 920 stress 0.3067014
## Run 921 stress 0.3089636
## Run 922 stress 0.3081541
## Run 923 stress 0.3075033
## Run 924 stress 0.3062996
## ... Procrustes: rmse 0.02598483 max resid 0.1733595
## Run 925 stress 0.3085003
## Run 926 stress 0.3062864
## ... Procrustes: rmse 0.02224969 max resid 0.1669829
## Run 927 stress 0.3066185
## Run 928 stress 0.3113644
## Run 929 stress 0.3073123
## Run 930 stress 0.3116866
## Run 931 stress 0.3069402
## Run 932 stress 0.3092141
## Run 933 stress 0.3098322
## Run 934 stress 0.3068616
## Run 935 stress 0.3083004
## Run 936 stress 0.3076929
## Run 937 stress 0.308413
## Run 938 stress 0.3087414
## Run 939 stress 0.3116025
## Run 940 stress 0.3102747
## Run 941 stress 0.3062869
## ... Procrustes: rmse 0.01635074 max resid 0.1448593
## Run 942 stress 0.3119254
## Run 943 stress 0.3090414
## Run 944 stress 0.3073983
## Run 945 stress 0.3065916
## Run 946 stress 0.3072722
## Run 947 stress 0.3144708
## Run 948 stress 0.3090428
## Run 949 stress 0.3067431
## Run 950 stress 0.3169323
## Run 951 stress 0.3095677
## Run 952 stress 0.3102234
## Run 953 stress 0.3066026
## Run 954 stress 0.3104171
## Run 955 stress 0.3091198
## Run 956 stress 0.3062298
## ... Procrustes: rmse 0.01991456 max resid 0.1457519
## Run 957 stress 0.3067628
## Run 958 stress 0.3072699
## Run 959 stress 0.3107368
## Run 960 stress 0.3071878
## Run 961 stress 0.3067034
## Run 962 stress 0.3067766
## Run 963 stress 0.3114634
## Run 964 stress 0.3080868
## Run 965 stress 0.3072443
## Run 966 stress 0.3063138
## Run 967 stress 0.3122521
## Run 968 stress 0.3077091
## Run 969 stress 0.3068741
## Run 970 stress 0.3088821
## Run 971 stress 0.3105779
## Run 972 stress 0.3095045
## Run 973 stress 0.3074448
## Run 974 stress 0.308065
## Run 975 stress 0.3104223
## Run 976 stress 0.3109977
## Run 977 stress 0.3079574
## Run 978 stress 0.3070857
## Run 979 stress 0.3091127
## Run 980 stress 0.3069742
## Run 981 stress 0.3070036
## Run 982 stress 0.3080799
## Run 983 stress 0.3083134
## Run 984 stress 0.3081597
## Run 985 stress 0.3089901
## Run 986 stress 0.3062107
## ... Procrustes: rmse 0.01532296 max resid 0.158084
## Run 987 stress 0.3074484
## Run 988 stress 0.3095566
## Run 989 stress 0.3085481
## Run 990 stress 0.3082917
## Run 991 stress 0.3078097
## Run 992 stress 0.3082252
## Run 993 stress 0.3064458
## Run 994 stress 0.3082021
## Run 995 stress 0.3086901
## Run 996 stress 0.3094554
## Run 997 stress 0.3110378
## Run 998 stress 0.3075698
## Run 999 stress 0.3072057
## Run 1000 stress 0.3074174
## *** No convergence -- monoMDS stopping criteria:
## 249: no. of iterations >= maxit
## 751: stress ratio > sratmax

ord

##
## Call:
## metaMDS(comm = SonD, distance = "bray", k = 2, try = 10, trymax = 1000, autotransform = TRUE, expand = TRUE, plot = FALSE)
##
## global Multidimensional Scaling using monoMDS
##
## Data: wisconsin(sqrt(SonD))
## Distance: bray
##
## Dimensions: 2
## Stress: 0.305813
## Stress type 1, weak ties
## No convergent solutions - best solution after 1000 tries
## Scaling: centring, PC rotation, halfchange scaling
## Species: expanded scores based on 'wisconsin(sqrt(SonD))'

site.scrs <- as.data.frame(scores(ord, display = "sites"))
site.scrs <- cbind(site.scrs, Site = SonDate$Site)

#Add environmental factors
Env <-SonDate[,5:10]
ef <- envfit(ord, Env, permutations = 999)
ef

##
## ***VECTORS
##
## NMDS1 NMDS2 r2 Pr(>r)
## MaxT 0.02562 -0.99967 0.0086 0.325
## MeanT -0.12407 -0.99227 0.0212 0.064 .
## MinT -0.23085 -0.97299 0.0220 0.061 .
## Elevation -0.31760 0.94822 0.0007 0.912
## AnnualPrecip -0.74166 0.67077 0.0057 0.468
## MonthlyPrecip 0.22886 0.97346 0.0391 0.008 **
## ---
## Signif. codes: 0 '***' 0.001 '**' 0.01 '*' 0.05 '.' 0.1 ' ' 1
## Permutation: free
## Number of permutations: 999

env.scores.dune <- as.data.frame(scores(ef, display = "vectors")) #extracts relevant scores from envifit
env.scores.dune <- cbind(env.scores.dune, env.variables = rownames(env.scores.dune)) #and then gives them their names
env.scores.dune <- cbind(env.scores.dune, pval = ef$vectors$pvals)
sig.env.scrs <- subset(env.scores.dune, pval<=0.1)

#Plot ordination
nmds.plot.SEGA <- ggplot(site.scrs, aes(x=NMDS1, y=NMDS2)) +
 geom_point(aes(NMDS1, NMDS2, colour = factor(SonDate$Treatment)))
nmds.plot.SEGA


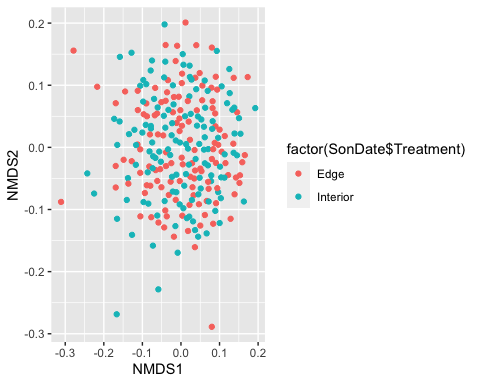


nmds.plot.SEGA+
 geom_segment(data = sig.env.scrs, aes(x = 0, xend=NMDS1, y=0, yend=NMDS2), arrow = arrow(length = unit(0.25, "cm")), colour = "grey10", lwd=0.5) + #add vector arrows of significant env variables
 ggrepel::geom_text_repel(data = sig.env.scrs, aes(x=NMDS1, y=NMDS2, label = env.variables), cex = 4, direction = "both", segment.size = 0.25)


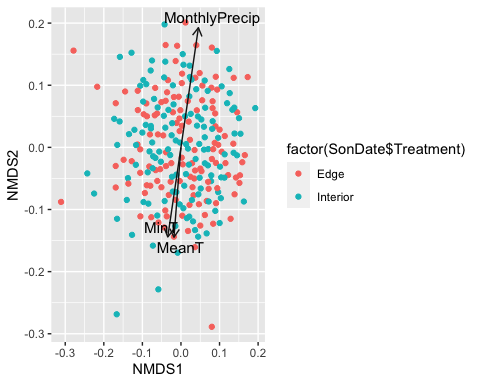


#Generalized Linear Models for Arthropod Variables, Part 1: setting up datasheet/variables

#LOAD DATASHEET: arthropod variables
Songlm <- read.csv("Library/Mobile Documents/com~apple~CloudDocs/Academia/NAU/Research/PhD/SonoranCommunity/glmm.csv", header = TRUE)

#MAKE VARAIBLES
#scale variables to eachother
En <- Songlm[,6:25]
En1 <- scale(En)
#create predictor variables
S <-Songlm$Site
D <-Songlm$Date
Treat <-Songlm$Treatment
Temp <-En$MeanT
Temp <- Temp/10
P <-En$MonthlyPrecip
Traps <-Songlm$Traps
S <- Songlm$Season

#Generalized Linear Models for Arthropod Variables, Part 2: building glmms for different arthropod response variables

#Change variable "R" to build different models for different arthropod response variables
#R <-En$Abundance
#R <- En$Richness
#R <-En$Mites
#R <-En$Spiders
#R <-En$TrueBugs
#R <-En$Springtails
#R <-En$Ant.Abundance
#R <-En$Beetle.Abundance
#R <-En$Bristletails
#R <-En$FireAnt

#For example: Arthropod Richness
R <- En$Richness
#Build null models with Poisson and negative binomoal distributions
RP = glmmTMB(R ~ 1 + offset(log(Traps)),
 ziformula = ~0,
 family = poisson(link = "log"),
 data = Songlm)
RNB = glmmTMB(R ~ 1 + offset(log(Traps)),
 ziformula = ~0,
 family = nbinom2(link = "log"),
 data = Songlm)
#Compare models, select best distribution based on AIC
anova(RP, RNB)

## Data: Songlm
## Models:
## RP: R ~ 1 + offset(log(Traps)), zi=~0, disp=~1
## RNB: R ~ 1 + offset(log(Traps)), zi=~0, disp=~1
## Df AIC BIC logLik deviance Chisq Chi Df Pr(>Chisq)
## RP 1 3443.2 3446.8 -1720.6 3441.2
## RNB 2 2121.4 2128.5 -1058.7 2117.4 1323.8 1 < 2.2e-16 ***
## ---
## Signif. codes: 0 '***' 0.001 '**' 0.01 '*' 0.05 '.' 0.1 ' ' 1

#Build a model saturated with predictor variables
R1 = glmmTMB(R ~ Treat * S + P + Temp + offset(log(Traps)),
 ziformula = ~0,
 family = nbinom2(link = "log"),
 data = Songlm,
 na.action = na.fail)
#Find best model with backwards stepwise elimination
best.model <- step(R1, direction = "backward", trace = 0, scope = list(lower = ~1, upper = ~ Treat * S + P + Temp + offset(log(Traps))), k = 2)
#Test best model for overdispersion and fit
simulationOutput <- simulateResiduals(fittedModel = best.model, n = 250)
plotSimulatedResiduals(simulationOutput = simulationOutput)

## plotSimulatedResiduals is deprecated, please switch your code to simply using the plot() function


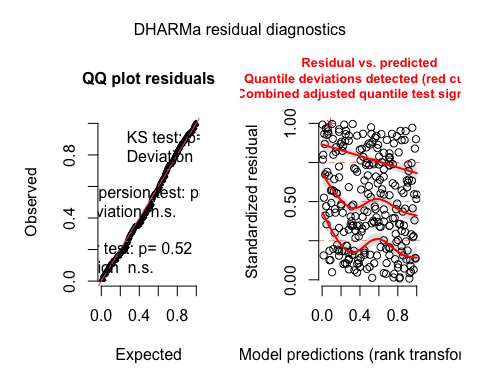


summary(best.model)

## Family: nbinom2 ( log )
## Formula: R ~ Treat + S + P + Treat:S + offset(log(Traps))
## Data: Songlm
##
## AIC BIC logLik deviance df.resid
## 2120.5 2156.0 -1050.2 2100.5 247
##
##
## Dispersion parameter for nbinom2 family (): 4.43
##
## Conditional model:
## Estimate Std. Error z value Pr(>|z|)
## (Intercept) 0.989422 0.104548 9.464 <2e-16 ***
## TreatInterior 0.277464 0.141048 1.967 0.0492 *
## SSpring 0.244880 0.131365 1.864 0.0623 .
## SSummer -0.046131 0.150117 -0.307 0.7586
## SWinter 0.107152 0.129647 0.826 0.4085
## P 0.018150 0.008393 2.162 0.0306 *
## TreatInterior:SSpring -0.457324 0.181719 -2.517 0.0118 *
## TreatInterior:SSummer -0.008900 0.208732 -0.043 0.9660
## TreatInterior:SWinter -0.146939 0.180097 -0.816 0.4146
## ---
## Signif. codes: 0 '***' 0.001 '**' 0.01 '*' 0.05 '.' 0.1 ' ' 1

#best model output
# Extract the coefficient table from the summary
summary_table <- summary(best.model)$coefficients
# Convert summary table to data frame
df_summary <- do.call(rbind, summary_table)
# View data frame
df_summary

## Estimate Std. Error z value Pr(>|z|)
## (Intercept) 0.989421890 0.104548079 9.46379786 2.969570e-21
## TreatInterior 0.277464487 0.141048475 1.96715695 4.916512e-02
## SSpring 0.244880387 0.131364836 1.86412433 6.230425e-02
## SSummer -0.046130732 0.150117457 -0.30729759 7.586169e-01
## SWinter 0.107151572 0.129647286 0.82648527 4.085289e-01
## P 0.018149950 0.008393392 2.16240951 3.058663e-02
## TreatInterior:SSpring -0.457323801 0.181718668 -2.51665833 1.184737e-02
## TreatInterior:SSummer -0.008899925 0.208731507 -0.04263815 9.659900e-01
## TreatInterior:SWinter -0.146938577 0.180096857 -0.81588640 4.145651e-01

# Convert the table to a dataframe
summary_df <- as.data.frame(df_summary)
summary_df$RowNames <- rownames(summary_df)
#Write to excel document
#write_xlsx(summary_df,"Library/Mobile Documents/com~apple~CloudDocs/Academia/NAU/Research/PhD/SonoranCommunity/Arthropod1.csv")
#if categorical variable (treatment/season) significant, use "emmeans(best.model, specs = pairwise ~ Treat|S)" for pairwise comparison

#CHAO analysis
SonChao <- read.csv("Library/Mobile Documents/com~apple~CloudDocs/Academia/NAU/Research/PhD/SonoranCommunity/SonoranChao.csv", header = TRUE)
SonChao <-SonChao[2:11]
DataInfo(SonChao, datatype="incidence_freq")

## site T U S.obs SC Q1 Q2 Q3 Q4 Q5 Q6 Q7 Q8 Q9 Q10
## 1 Bell 288 1951 128 0.9755 48 15 14 5 4 3 5 1 2 2
## 2 Dixileta 264 2033 131 0.9814 38 22 11 8 8 0 6 1 6 3
## 3 Gateway 295 1979 147 0.9773 45 22 10 10 6 8 4 5 5 3
## 4 LoneMtn 240 3050 148 0.9847 47 29 10 6 8 4 5 5 3 2
## 5 Mine 111 2368 102 0.9849 36 16 12 7 6 3 0 2 0 1
## 6 Paraiso 282 3339 130 0.9899 34 22 10 6 8 8 4 3 7 2
## 7 Prospector 108 662 92 0.9462 36 19 8 2 5 2 3 1 3 1
## 8 Rincon 286 2876 140 0.9844 45 19 7 9 7 7 6 1 2 1
## 9 Sunrise 282 2403 143 0.9809 46 27 11 7 6 3 1 3 2 2
## 10 TomThumb 303 3021 154 0.9822 54 18 18 12 5 4 2 3 3 3

estimateD(SonChao, datatype="abundance", base= "size", level=2000)

## site m method order SC qD qD.LCL qD.UCL
## 1 Bell 2000 interpolated 0 0.977 123.688 114.253 133.124
## 2 Bell 2000 interpolated 1 0.977 28.042 26.234 29.851
## 3 Bell 2000 interpolated 2 0.977 14.953 14.022 15.885
## 4 Dixileta 2000 interpolated 0 0.981 126.694 118.827 134.561
## 5 Dixileta 2000 interpolated 1 0.981 30.926 29.037 32.816
## 6 Dixileta 2000 interpolated 2 0.981 15.305 14.312 16.297
## 7 Gateway 2000 interpolated 0 0.978 142.240 133.219 151.260
## 8 Gateway 2000 interpolated 1 0.978 36.725 34.404 39.047
## 9 Gateway 2000 interpolated 2 0.978 17.633 16.367 18.899
## 10 LoneMtn 2000 interpolated 0 0.977 125.273 118.427 132.119
## 11 LoneMtn 2000 interpolated 1 0.977 24.845 23.507 26.183
## 12 LoneMtn 2000 interpolated 2 0.977 12.087 11.337 12.838
## 13 Mine 2000 interpolated 0 0.982 95.350 87.706 102.994
## 14 Mine 2000 interpolated 1 0.982 13.805 12.894 14.717
## 15 Mine 2000 interpolated 2 0.982 6.545 6.153 6.937
## 16 Paraiso 2000 interpolated 0 0.982 110.003 104.174 115.831
## 17 Paraiso 2000 interpolated 1 0.982 23.474 22.477 24.472
## 18 Paraiso 2000 interpolated 2 0.982 13.276 12.718 13.835
## 19 Prospector 2000 extrapolated 0 0.991 120.758 101.240 140.277
## 20 Prospector 2000 extrapolated 1 0.991 30.995 27.833 34.158
## 21 Prospector 2000 extrapolated 2 0.991 15.491 13.751 17.232
## 22 Rincon 2000 interpolated 0 0.980 121.318 113.771 128.866
## 23 Rincon 2000 interpolated 1 0.980 28.200 26.756 29.645
## 24 Rincon 2000 interpolated 2 0.980 13.852 12.965 14.740
## 25 Sunrise 2000 interpolated 0 0.977 130.290 122.865 137.715
## 26 Sunrise 2000 interpolated 1 0.977 29.249 27.526 30.972
## 27 Sunrise 2000 interpolated 2 0.977 14.015 13.130 14.899
## 28 TomThumb 2000 interpolated 0 0.976 129.127 121.975 136.280
## 29 TomThumb 2000 interpolated 1 0.976 26.100 24.706 27.494
## 30 TomThumb 2000 interpolated 2 0.976 12.088 11.321 12.854
