# Supplementary material for: Living on the edge: The sensitivity of arthropods to development and climate along an urban-wildland interface in the Sonoran Desert of central Arizona
Source: PLoS One. 2024 Apr 18;19(4):e0297227. doi: 10.1371/journal.pone.0297227 (PMC11025936; doi:10.1371/journal.pone.0297227)
Supplement: Attachment — Submitted filename: PONE-D-23-29401_Review_v1.docx [file pone.0297227.s005.docx]

In this study, the authors compared ground-dwelling arthropod community composition between edge and interior sites of a Preserve partially surrounded by urban areas. I believe the collected data is valuable, and the question regarding whether the effects of urbanization on arthropod communities spill over into adjacent natural areas is highly relevant. Nevertheless, I think that the study’s focus should be more explicit, and the hypotheses/expectations should be better justified. All my comments are detailed below.

- Lines 18-19 (Abstract): The terms "arthropod dynamics" refers to the changes in arthropod populations or communities over time. Because this study did not measure temporal variations in arthropod communities, I find this sentence misleading.
- Lines 49-58 (Introduction): It is not clear to me why the spill over effect would be different in arid regions. I think that this paragraph should better highlight this point in order to justify the novelty of this study. What are the hypotheses/expectations? Would the spill over be stronger because the effect of urbanization on community composition is more pronounced in arid areas than in temperate areas?
- Lines 65-71 (Introduction): I think that the hypotheses/expectations should be better justified. The knowledge gap highlighted lines 59-60 is about the effect of urbanization on arthropod communities spilling over the surrounding natural area. Yet, several hypotheses/expectations are about climate. Moreover, why do the authors expect an interaction between climate and habitat? In addition, edge habitats are usually richer in biodiversity. Why in this case the authors expect the reverse?
- Lines 89-90 (Methods): Even though this part of the boundary was not adjacent to a developed area, it does not seem correct to write that both sites are on the interior of the Preserve. It should probably be written that both sites have been classified in the treatment "interior".
- Line 121 (Methods): Based on the study design described above, it should be four edge sites and six interior sites.
- Line 130 (Methods): I understand by “we treated empty traps as zero-samples in analyse” that "0" was entered in the community dataset for all the arthropod groups at this sampling point. Is that correct?
- Lines 185-187 (Methods): It is not clear to me what variables were added as random intercepts.
- Lines 208-209 (Methods): I understand that the authors run a MDS (Metric Multidimentional Scaling); is that correct? Did the authors considered using a partial transformation based redundancy analysis with the location (the five pairs), and maybe also the year, as covariates? In my opinion, I think this approach would be more appropriate to test the effect of the urban edge (as well as season, temperature and precipitation) on the variation in community composition.
- Figure 2 (Results): The figures 2C and 2D would be easier to read if background colours would be used for each season and only the year would be displayed on the X axis. This could also be implemented in figures 1B and 1C.
- Lines 237-238 (Results): Because not everyone knows the Hill numbers by heart, I think that it would be easier for the reader if the diversity indices measured would be displayed instead of referring to the Hill number.
- Figure 2 (Results): Same thing here, I would suggest to replace in Figure 3 the Hill numbers by the diversity index measured.
- Lines 241-242 (Results): I would suggest writing, "the pair for which both sites were classified as interior".
- Line 255 (Results): "were shared"?
- Figure 4 (Results): The information provided by Figure 4D is not particularly informative. I would suggest using symbols for interior/edge treatments, and colours for the five site pairs. Moreover, I think that a partial RDA might be more appropriate (see previous comment).
- Lines 294-300 (Results): It would be more informative to write down the direction of the associations between alpha diversity and climate, and to indicate the percentage of the variation explained by these variables (for instance using the 'r2_nakagawa' function). Moreover, Figure 4 does not show the data on taxonomic groups, which makes it impossible to understand how temperature and humidity shape arthropod community composition.
- Table 3 (Results): A table combining Table 3 and Table S4 would be more informative (see example below).

|  | **Treatment** | **Season** | **Precipitation** | **Temperature** | **Treatment x Season** | **R²** |
| --- | --- | --- | --- | --- | --- | --- |
| **Arthropod abundance** | F = x; p = y |  |  |  |  |  |
| **Arthropod LPTs** |  |  |  |  |  |  |
| **Ant abundance** |  |  |  |  |  |  |
| **Beetle abundance** |  |  |  |  |  |  |
| **Bristletail abundance** |  |  |  |  |  |  |
| **Mite abundance** |  |  |  |  |  |  |
| **Spider abundance** |  |  |  |  |  |  |
| **True bug abundance** |  |  |  |  |  |  |
| **Springtail abundance** |  |  |  |  |  |  |
| **Fire ant abundance** |  |  |  |  |  |  |

- Figure 5 (Results): In Figure 5F, the asterisk seems to be missing.
- Lines 329-332 (Discussion): To me, the main conclusions of this study is that there is no consistent effect of urban edge on the composition of arthropod communities. The effects observed are site, season and taxon - specific.
- Lines 360-370 (Discussion): There is a rather big part of the result section aiming at highlighting groups that were found exclusively in the interior or the edge treatment. Therefore, I think that it would be worth adding to this paragraph a discussion about these results. Are the taxa specific to the urban edge also common in urban areas? Are they native? Are there specificity in their ecology that would explain that they are found in one treatment and not the other?
- Lines 383-384 (Discussion): Does the urban heat island expend to the Preserve edges? If not, this comment is probably not relevant.
- Line 385 (Discussion): I understood from Table 1 that the vegetation is mainly composed of shrubs. Moreover, from the pictures, there seem to be no or few trees in the natural area. Therefore, planting trees seems to me like altering even further the ecosystem at the urban edge.
