# Supplementary material for: Living on the edge: The sensitivity of arthropods to development and climate along an urban-wildland interface in the Sonoran Desert of central Arizona
Source: PLoS One. 2024 Apr 18;19(4):e0297227. doi: 10.1371/journal.pone.0297227 (PMC11025936; doi:10.1371/journal.pone.0297227)
Supplement: Attachment — Submitted filename: Response to Reviewers.docx [file pone.0297227.s006.docx]

**Response to Reviewers**

PONE-D-23-29401

Living on the edge: The sensitivity of arthropods to development and climate along an urban-wildland interface in the Sonoran Desert of central Arizona

We thank the editor(s) and reviewers for their valuable feedback which has helped us improve our manuscript. We have included a tracked-change file documenting the changes we’ve made in our manuscript, our final manuscript, and below our responses to reviewers in blue text.

**Journal Requirements:**

When submitting your revision, we need you to address these additional requirements.

1. Please ensure that your manuscript meets PLOS ONE's style requirements, including those for file naming. The PLOS ONE style templates can be found at
   <https://journals.plos.org/plosone/s/file?id=wjVg/PLOSOne_formatting_sample_main_body.pdf> and 
   <https://journals.plos.org/plosone/s/file?id=ba62/PLOSOne_formatting_sample_title_authors_affiliations.pdf>

Thank you, we have formatted our manuscript for PLOS ONE.

2. Note from Emily Chenette, Editor in Chief of PLOS ONE, and Iain Hrynaszkiewicz, Director of Open Research Solutions at PLOS: Did you know that depositing data in a repository is associated with up to a 25% citation advantage (<https://doi.org/10.1371/journal.pone.0230416>)? If you’ve not already done so, consider depositing your raw data in a repository to ensure your work is read, appreciated and cited by the largest possible audience. You’ll also earn an Accessible Data icon on your published paper if you deposit your data in any participating repository (<https://plos.org/open-science/open-data/#accessible-data>).

We thank the editor in chief for the suggestion, but have our data available on a pubically accessible repository already (cited in our paper).

3. In your Methods section, please provide additional information regarding the permits you obtained for the work. Please ensure you have included the full name of the authority that approved the field site access and, if no permits were required, a brief statement explaining why.

We have added a statement into the methods, “Permits for arthropod collection were obtained from the City of Scottsdale through the McDowell Sonoran Preserve.”

4. We note that you have stated that you will provide repository information for your data at acceptance. Should your manuscript be accepted for publication, we will hold it until you provide the relevant accession numbers or DOIs necessary to access your data. If you wish to make changes to your Data Availability statement, please describe these changes in your cover letter and we will update your Data Availability statement to reflect the information you provide.

Our data is already pubically accessible, we do not wish to provide the data to another repository.

5. Please include your full ethics statement in the ‘Methods’ section of your manuscript file. In your statement, please include the full name of the IRB or ethics committee who approved or waived your study, as well as whether or not you obtained informed written or verbal consent. If consent was waived for your study, please include this information in your statement as well.

Our study requires no ethics statement.

6. We note that Figure 1 in your submission contain [map/satellite] images which may be copyrighted. All PLOS content is published under the Creative Commons Attribution License (CC BY 4.0), which means that the manuscript, images, and Supporting Information files will be freely available online, and any third party is permitted to access, download, copy, distribute, and use these materials in any way, even commercially, with proper attribution. For these reasons, we cannot publish previously copyrighted maps or satellite images created using proprietary data, such as Google software (Google Maps, Street View, and Earth). For more information, see our copyright guidelines: <http://journals.plos.org/plosone/s/licenses-and-copyright>.
 
We require you to either (1) present written permission from the copyright holder to publish these figures specifically under the CC BY 4.0 license, or (2) remove the figures from your submission:
 
a. You may seek permission from the original copyright holder of Figure 1 to publish the content specifically under the CC BY 4.0 license.  
 
We recommend that you contact the original copyright holder with the Content Permission Form (<http://journals.plos.org/plosone/s/file?id=7c09/content-permission-form.pdf>) and the following text:
“I request permission for the open-access journal PLOS ONE to publish XXX under the Creative Commons Attribution License (CCAL) CC BY 4.0 (<http://creativecommons.org/licenses/by/4.0/>). Please be aware that this license allows unrestricted use and distribution, even commercially, by third parties. Please reply and provide explicit written permission to publish XXX under a CC BY license and complete the attached form.”
 
Please upload the completed Content Permission Form or other proof of granted permissions as an ""Other"" file with your submission.
 
In the figure caption of the copyrighted figure, please include the following text: “Reprinted from [ref] under a CC BY license, with permission from [name of publisher], original copyright [original copyright year].”
 
b. If you are unable to obtain permission from the original copyright holder to publish these figures under the CC BY 4.0 license or if the copyright holder’s requirements are incompatible with the CC BY 4.0 license, please either i) remove the figure or ii) supply a replacement figure that complies with the CC BY 4.0 license. Please check copyright information on all replacement figures and update the figure caption with source information. If applicable, please specify in the figure caption text when a figure is similar but not identical to the original image and is therefore for illustrative purposes only.
The following resources for replacing copyrighted map figures may be helpful:
 
USGS National Map Viewer (public domain): <http://viewer.nationalmap.gov/viewer/>
The Gateway to Astronaut Photography of Earth (public domain): <http://eol.jsc.nasa.gov/sseop/clickmap/>
Maps at the CIA (public domain): <https://www.cia.gov/library/publications/the-world-factbook/index.html> and <https://www.cia.gov/library/publications/cia-maps-publications/index.html>
NASA Earth Observatory (public domain): <http://earthobservatory.nasa.gov/>
Landsat: <http://landsat.visibleearth.nasa.gov/>
USGS EROS (Earth Resources Observatory and Science (EROS) Center) (public domain): [http://eros.usgs.gov/#](http://eros.usgs.gov/)
Natural Earth (public domain): <http://www.naturalearthdata.com/>

We have removed the satellite imagery in figure 1.

7. Please review your reference list to ensure that it is complete and correct. If you have cited papers that have been retracted, please include the rationale for doing so in the manuscript text, or remove these references and replace them with relevant current references. Any changes to the reference list should be mentioned in the rebuttal letter that accompanies your revised manuscript. If you need to cite a retracted article, indicate the article’s retracted status in the References list and also include a citation and full reference for the retraction notice.

We have reviewed our references. Only one reference changed during revisions, #13 was changed.

**Reviewer #1:**

In this study, the authors compared ground-dwelling arthropod community composition between edge and interior sites of a Preserve partially surrounded by urban areas. I believe the collected data is valuable, and the question regarding whether the effects of urbanization on arthropod communities spill over into adjacent natural areas is highly relevant. Nevertheless, I think that the study’s focus should be more explicit, and the hypotheses/expectations should be better justified. All my comments are detailed below.

We thank the reviewer for their helpful insights which have improved our manuscript. We have worked to make the hypotheses better justified.

- Lines 18-19 (Abstract): The terms "arthropod dynamics" refers to the changes in arthropod populations or communities over time. Because this study did not measure temporal variations in arthropod communities, I find this sentence misleading.

We understand the reviewers concern and have changed “dynamics” to “communities”

- Lines 49-58 (Introduction): It is not clear to me why the spill over effect would be different in arid regions. I think that this paragraph should better highlight this point in order to justify the novelty of this study. What are the hypotheses/expectations? Would the spill over be stronger because the effect of urbanization on community composition is more pronounced in arid areas than in temperate areas?

We have clarified our language here to demonstrate why arthropods in arid climates are likely to be especially affected by urbanization. Specifically, the urban heat effect can raise temperature and change precipitation patterns, with effects extending into naturalized areas. Therefore we’d expect that arthropods on the urban-edge would be more climate stressed than arthropods on the urban edge of other systems (e.g. temperate areas).

- Lines 65-71 (Introduction): I think that the hypotheses/expectations should be better justified. The knowledge gap highlighted lines 59-60 is about the effect of urbanization on arthropod communities spilling over the surrounding natural area. Yet, several hypotheses/expectations are about climate. Moreover, why do the authors expect an interaction between climate and habitat? In addition, edge habitats are usually richer in biodiversity. Why in this case the authors expect the reverse?

We have clarified in our introduction why we expect an interaction between climate and urbanization in their effects on arthropods. Urbanization alters climate with spill over effects into urban edges, making these areas even more environmentally stressful than normal. Certain seasons with the most environmental stress are likely to produce the most profound effects on arthropod communities, such as we find in this study.

We hypothesized that arthropods would have lower richness on the urban edge because several studies in our region found arthropods had lower richness in urbanized areas compared to naturalized areas (Bang & Faeth 2011, Cook & Faeth 2006).

Bang C, Faeth SH. Variation in arthropod communities in response to urbanization: seven years of arthropod monitoring in a desert city. Landsc Urban Plan. 2011;103(3-4):383-399.

Cook WM, Faeth SH. Irrigation and land use drive ground arthropod community patterns in an urban desert. Environ Entomol. 2006;35(6):1532-1540.

- Lines 89-90 (Methods): Even though this part of the boundary was not adjacent to a developed area, it does not seem correct to write that both sites are on the interior of the Preserve. It should probably be written that both sites have been classified in the treatment "interior".

Agreed, we have changed our language based on the reviewer’s suggestion.

- Line 121 (Methods): Based on the study design described above, it should be four edge sites and six interior sites.

Thank you, we have corrected this.

- Line 130 (Methods): I understand by “we treated empty traps as zero-samples in analyse” that "0" was entered in the community dataset for all the arthropod groups at this sampling point. Is that correct?

Yes, that is correct.

- Lines 185-187 (Methods): It is not clear to me what variables were added as random intercepts.

We clarified that trap number was the random variable.

- Lines 208-209 (Methods): I understand that the authors run a MDS (Metric Multidimentional Scaling); is that correct? Did the authors considered using a partial transformation based redundancy analysis with the location (the five pairs), and maybe also the year, as covariates? In my opinion, I think this approach would be more appropriate to test the effect of the urban edge (as well as season, temperature and precipitation) on the variation in community composition.

Yes, we ran a MDS as stated in our methods. We tested a partial transformation based redundancy analysis with location as the covariate (among other approaches) as the reviewer suggested. However, we found the similar (non)results as the MDS approach. Specifically, the conditional and constrained proportions of the RDAs only added up to ~4%, leaving ~96% of the variation unexplained. This is similar to our non-significant findings of the MDS, except with the MDS method we did find effects of temperature and precipitation which are helpful in explaining arthropod community patterns. Therefore the RDA approach does not add any value to our manuscript and we chosen to omit it.

- Figure 2 (Results): The figures 2C and 2D would be easier to read if background colours would be used for each season and only the year would be displayed on the X axis. This could also be implemented in figures 1B and 1C.

Thank you for the suggestion, we have implemented this in figure 2C and 2D, and figure 1B and 1C.

- Lines 237-238 (Results): Because not everyone knows the Hill numbers by heart, I think that it would be easier for the reader if the diversity indices measured would be displayed instead of referring to the Hill number.

We have taken the reviewer’s suggestion and edited our manuscript accordingly.

- Figure 2 (Results): Same thing here, I would suggest to replace in Figure 3 the Hill numbers by the diversity index measured.

We have taken the reviewer’s suggestion and edited Fig. 3 accordingly.

- Lines 241-242 (Results): I would suggest writing, "the pair for which both sites were classified as interior".

Thank you, we accepted this suggestion.

- Line 255 (Results): "were shared"?

Corrected.

- Figure 4 (Results): The information provided by Figure 4D is not particularly informative. I would suggest using symbols for interior/edge treatments, and colours for the five site pairs. Moreover, I think that a partial RDA might be more appropriate (see previous comment).

We have coded site pairs as colours and treatment as symbols as suggested. Above we show that the partial RDA gave the same (non)results of the MDS. While it is nice to see separation in ordinations, the lack of separation (nonsignificant grouping results) is good to visualize in our study. PLoS One publishes null results because these types of findings are still valuable to scientists; our lack of grouping in the MDS ordination shows that beta-diversity of arthropods is not well explained by site and treatment in our study. However, our MDS does visualize that secondary environmental variables (precipitation and temperature) are important for arthropod beta-diversity. Therefore, we consider Figure 4D to be informative enough to include.

- Lines 294-300 (Results): It would be more informative to write down the direction of the associations between alpha diversity and climate, and to indicate the percentage of the variation explained by these variables (for instance using the 'r2_nakagawa' function). Moreover, Figure 4 does not show the data on taxonomic groups, which makes it impossible to understand how temperature and humidity shape arthropod community composition.

We clarified in the results text that precipitation and temperature were positive predictors for arthropods. We have also added R2 values to Table 3.

- Table 3 (Results): A table combining Table 3 and Table S4 would be more informative (see example below).

|  | **Treatment** | **Season** | **Precipitation** | **Temperature** | **Treatment x Season** | **R²** |
| --- | --- | --- | --- | --- | --- | --- |
| **Arthropod abundance** | F = x; p = y |  |  |  |  |  |
| **Arthropod LPTs** |  |  |  |  |  |  |
| **Ant abundance** |  |  |  |  |  |  |
| **Beetle abundance** |  |  |  |  |  |  |
| **Bristletail abundance** |  |  |  |  |  |  |
| **Mite abundance** |  |  |  |  |  |  |
| **Spider abundance** |  |  |  |  |  |  |
| **True bug abundance** |  |  |  |  |  |  |
| **Springtail abundance** |  |  |  |  |  |  |
| **Fire ant abundance** |  |  |  |  |  |  |

We have added R^2^ values to Table 3. We are not sure which parts of Supp. Table 4 the reviewer is suggesting we add. There also appears to be some formatting error (likely because of Microsoft-Word’s table issues) in the table the reviewer provided. We chose to have model details in Supp. Table 4 because a) there is not room for all details in this table, b) p-values are not needed as we state “only significant (p<0.05) predictors are shown”, c) the model details would take away/bury the main message of the table, and d) anyone who is interested in checking the model details can see the referenced Supp. Table 4.

- Figure 5 (Results): In Figure 5F, the asterisk seems to be missing.

Corrected.

- Lines 329-332 (Discussion): To me, the main conclusions of this study is that there is no consistent effect of urban edge on the composition of arthropod communities. The effects observed are site, season and taxon - specific.

We agree and have added this language in the beginning of our discussion.

- Lines 360-370 (Discussion): There is a rather big part of the result section aiming at highlighting groups that were found exclusively in the interior or the edge treatment. Therefore, I think that it would be worth adding to this paragraph a discussion about these results. Are the taxa specific to the urban edge also common in urban areas? Are they native? Are there specificity in their ecology that would explain that they are found in one treatment and not the other?

We thank the reviewer for this suggestion and have added a paragraph into the discussion on highlighting the results of taxa found exclusively in either the interior or edge treatment.

- Lines 383-384 (Discussion): Does the urban heat island expend to the Preserve edges? If not, this comment is probably not relevant.

Yes it does, we have clarified that in our manuscript.

- Line 385 (Discussion): I understood from Table 1 that the vegetation is mainly composed of shrubs. Moreover, from the pictures, there seem to be no or few trees in the natural area. Therefore, planting trees seems to me like altering even further the ecosystem at the urban edge.

We agree, we’ve changed the suggest from “trees” to “native vegetation”

**Reviewer #2:**

We thank the reviewer for their valuable comments.

Lines 49-52: Authors reference that arthropods in arid regions adapt to hot and dry environments but do poorly in urban areas because of these adaptations. The authors need to add a sentence of justification for this since cities are often hotter and dryer than rural areas. Perhaps that these are already extreme environments and any additional perturbation makes them vulnerable. As it states now, I am not convinced.

We have edited this paragraph of the introduction to better justify why arthropod communities are more sensitive to urbanization in arid climates. Essentially, these are already extreme environments, and urbanization can enhance those extremes through the urban heat effect.

Lines 124-129: Was there anything at the bottom of the cup in the pitfall trap to keep insects from crawling or jumping out of the trap? Were they set out as dry? This will affect the types and counts of arthropods and while they would be consistent across sampling, it does bias some of the end results.

Cups were set out dry, with nothing in the bottom, we added a clarification of ‘empty plastic cup’ in our methods. This was consistent across sampling. We note that trap contents were similar to other studies in our region (e.g. Uhey et al. 2021, Uhey et al. 2022).

Lines 185-195: Did you also test for year? The samples were collected across multiple years and the methods do not state how the year of collection was handled. Were the seasons averaged across years or individual per year?

We found that the variability of the data across years was extreme, as arthropod community data often is, therefore any analysis testing ‘year’ did not work. We found the more ecologically relevant categorical variable of ‘season’ was more helpful in explaining arthropod community variability.

As stated in our methods, for most analyses we used each sample (pitfall trap at site/date), not averages (except the MDS-ordination, here we state “we used the average arthropod abundance of the individual pitfall traps for each unique sample date at each site”).

Table 2 would be helpful if there was an additional column that included native/invasive. Were the edge exclusive LPTs because they were non-natives associated with urban or were they species that typically do well in edge regions?

We believe these are all native species, but given only a handful of the LPTs are identified to species, we cannot be certain. Thus, we cannot add a column designating native/invasive with confidence. We have added these points into our discussion.
